# Design and Validation of a Custom Next-Generation Sequencing Panel in Pediatric Acute Lymphoblastic Leukemia

**DOI:** 10.3390/ijms24054440

**Published:** 2023-02-23

**Authors:** José Vicente Gil, Esperanza Such, Claudia Sargas, Javier Simarro, Alberto Miralles, Gema Pérez, Inmaculada de Juan, Sarai Palanca, Gayane Avetisyan, Marta Santiago, Carolina Fuentes, José María Fernández, Ana Isabel Vicente, Samuel Romero, Marta Llop, Eva Barragán

**Affiliations:** 1 Accredited Research Group on Hematology, Instituto de Investigación Sanitaria la Fe, 46026 Valencia, Spain; 2 Hematology Diagnostic Unit, Hematology Service, Hospital Universitario y Politécnico la Fe, 46026 Valencia, Spain; 3 Centro de Investigación Biomédica en Red de Cáncer, CIBERONC CB16/12/00284, Instituto de Salud Carlos III, 28029 Madrid, Spain; 4 Accredited Research Group on Clinical and Translational Cancer Research, Instituto de Investigación Sanitaria la Fe, 46026 Valencia, Spain; 5 Molecular Biology Unit, Clinical Analysis Service, Hospital Universitario y Politécnico la Fe, 46026 Valencia, Spain; 6 Department of Biochemistry and Molecular Biology, University of Valencia, 46010 Valencia, Spain; 7 Onco-Hematology Unit, Pediatrics Service, Hospital Universitario y Politécnico la Fe, 46026 Valencia, Spain

**Keywords:** next-generation sequencing, NGS, molecular characterization, childhood acute lymphoblastic leukemia

## Abstract

The molecular landscape of acute lymphoblastic leukemia (ALL) is highly heterogeneous, and genetic lesions are clinically relevant for diagnosis, risk stratification, and treatment guidance. Next-generation sequencing (NGS) has become an essential tool for clinical laboratories, where disease-targeted panels are able to capture the most relevant alterations in a cost-effective and fast way. However, comprehensive ALL panels assessing all relevant alterations are scarce. Here, we design and validate an NGS panel including single-nucleotide variants (SNVs), insertion–deletions (indels), copy number variations (CNVs), fusions, and gene expression (ALLseq). ALLseq sequencing metrics were acceptable for clinical use and showed 100% sensitivity and specificity for virtually all types of alterations. The limit of detection was established at a 2% variant allele frequency for SNVs and indels, and at a 0.5 copy number ratio for CNVs. Overall, ALLseq is able to provide clinically relevant information to more than 83% of pediatric patients, making it an attractive tool for the molecular characterization of ALL in clinical settings.

## 1. Introduction

Acute lymphoblastic leukemia (ALL) is the most common type of pediatric cancer. The current cure rate reaches 80–90%, but it decreases with age, and the prognosis of relapsed patients is very poor [1,2].

ALL is caused by the clonal proliferation of immature lymphocytes due to the accumulation of genetic alterations, which gives rise to different biological and clinical subtypes of leukemia. The presence of genetic lesions that drive distinct subtypes of leukemia has set the bases of ALL classification, in which novel categories defined by point mutations (such as *PAX5* p.P80R or *IKZF1* p.N159N) and additional gene fusions (i.e., *ZNF384* or *MEF2D* rearrangements) have been recently acknowledged [3,4]. Some of these variants, as well as copy number variation (CNV) affecting other genes, are used for risk stratification, which drives treatment intensity [5,6]. Genetic lesions can further be utilized for targeted therapy selection, against either the affected gene or the altered signaling pathway [7,8]. Therefore, as molecular variants are used as diagnostic, prognostic, and predictive biomarkers, their identification is being increasingly demanded by pediatricians and hematologists.

Next-generation sequencing (NGS) allows the simultaneous assessment of a broad number of targets in several samples and has therefore become an indispensable tool for clinical laboratories. Different approaches have been developed in order to optimize resources and shorten the turnaround time, with pathology-directed panels being the preferred option in most centers [9]. However, the availability of commercial ALL NGS panels is scarce, as pan-hematological assays usually lack relevant genes or omit CNV identification.

The purpose of this work was to design and validate an ALL-targeted NGS panel (ALLseq), including clinically relevant point mutations, insertion–deletions (indels), CNVs, fusions, and gene expression. Our results show that ALLseq constitutes a useful tool for patient characterization, identifying driver and secondary alterations in a single experiment, thus allowing accurate diagnosis, patient risk stratification, and (in some cases) treatment selection.

## 2. Results

### 2.1. ALLseq Design

The ALLseq design included the targets listed in Table 1 and Supplemental Table S1, for which point mutations, indels, CNVs, fusions, and/or gene expression can be assessed.

### 2.2. ALLseq Set-Up and Sequencing Metrics

The maximum chip efficiency was obtained when 200 ng of the library pool (4:1 DNA:RNA) was used for template preparation. Under these conditions, the mean chip load was 88.3% (35.88% polyclonal; 55.6% usable reads), which yielded a mean of 18,451,079 total reads.

The mean quality metrics per sample showed a read depth of 1903×, on-target and uniformity percentages > 95%, and a median absolute pairwise difference (MAPD) mean value of 0.19. The mean read depth < 100× was found on 9/1138 amplicons (0.8%).

**Figure 1:**
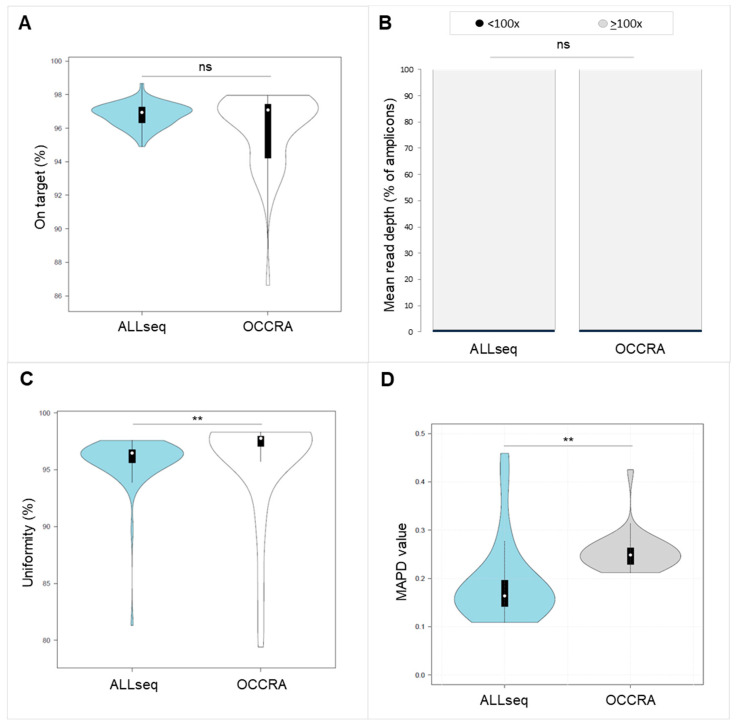
Comparison of sequencing metrics between ALLseq and OCCRA panels. (**A**) On-target percentage. (**B**) Mean number of amplicons showing a mean read depth < 100×. (**C**) Uniformity percentage. (**D**) MAPD. ns = not significant; ** indicates *p* < 0.05.

We compared the ALLseq main sequencing metrics with those obtained with the Oncomine Childhood Research Assay (OCCRA). The OCCRA is a commercial pan-pediatric cancer panel, where some ALL targets are present. No significant differences were found for the on-target % or the percentage of amplicons showing a low mean read depth (<100×) (Figure 1A,B). However, the median uniformity % and MAPD were lower in ALLseq (uniformity: 96.46 vs. 97.77, *p* < 0.05; MAPD 0.16 vs. 0.25) (Figure 1C,D).

Focusing on RNA results, the mean total number of RNA mapped reads per sample was 358,240. All of the analyzed samples passed the quality control test (>20,000 total mapped reads).

### 2.3. Technical Validation Strategy and ALLseq Performance

For technical validation, 25 molecularly characterized samples were selected. Nineteen carried SNVs or indels in *DNM2, EP300, FBXW7, FLT3, H3F3A, IKZF1, JAK1, JAK3, KMT2D, NOTCH1, NRAS, PHF6, PTPN11*, or *TP53*. Eighteen samples harbored *CDKN2A, CDKN2B, IKZF1, JAK2, RB1, PAX5*, and/or *ETV6* deletions. Six samples were used as DNA negative controls for SNVs/indels, and seven for CNVs.

Seventeen RNA samples carried one of the following fusions: *TCF3::ZNF384, KMT2A::MLLT10, BCR::ABL1, TCF3::PBX1, ARID1B::ZNF384, PICALM::MLLT10, TERF::JAK2, IGH::CRLF2* (does not generate a fusion transcript but overexpresses CRLF2), *ETV6::RUNX1, KMT2A::AFF1, STIL::TAL1* (overexpresses TAL1), or *EBF1::PDGFRB*. Eight samples were used as negative RNA controls.

#### 2.3.1. SNVs and Indels

**Figure 2:**
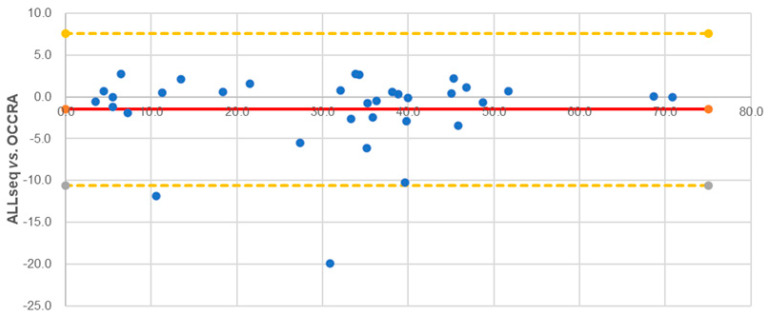
Bland–Altman representation of VAF values obtained by ALLseq and OCCRA panels.

ALLseq detected all of the expected SNVs and indels; *KMT2D* c.8743C>T and *H3F3A* c.82A>G were not detected, as these genes were not included in the ALLseq design. ALLseq identified two additional variants: *DNM2* c.2080G>T (not included in OCCRA’s design) and *KRAS* c.34G>C (confirmed by direct sequencing and Minor Variant Finder analysis) (Supplemental Table S2). The VAF of the 34 overlapping variants showed a high correlation between ALLseq and OCCRA (R^2^ = 0.93), and the Bland-Altman plot showed that 93.9% of the VAF values were in agreement within the 95% confidence intervals (Figure 2).

#### 2.3.2. CNVs

ALLseq detected at least one CNV in 18/25 (72%) samples and a total of 39 CNVs: *CDKN2A*—9/39 (23.1%), *CDKN2B*—9/39 (23.1%), *ETV6*—6/39 (15.4%), *PAX5*—6/39 (15.4%), *IKZF1*—4/39 (10.3%), *JAK2*—2/39 (5.1%), *RB1*—2/39 (5.1%), and *EBF1*—1/39 (2.6%). Eight discrepancies were observed between ALLseq and MLPA (three for *IKZF1*, two for *PAX5*, one for *ETV6*, one for *EBF1*, and one for *RB1*) (Supplemental Table S3). Altogether, the CNV Cohen’s kappa coefficient was 0.88.

#### 2.3.3. Fusions and Gene Expression

ALLseq detected fusions and/or high gene expression in 18/25 samples (72%). No false positives were observed (Supplemental Table S4).

RNA 10 showed *CRLF2* expression 229-fold higher than the median, consistent with the presence of a *CRLF2* translocation at the IGH locus in this sample. Similarly, RNA 18, which harbored a *STIL::TAL1* fusion (also identified by ALLseq), expressed *TAL1* 25-fold compared to the median (Supplemental Table S5). The expression values of *CRLF2* and *TAL1* measured by ALLseq and RT-qPCR showed a high correlation (R^2^ = 0.98 and 0.90, respectively) (Supplemental Figure S1). As expected, *TLX1*, *TLX3*, *NKX2-1*, and *HOXAA* expressions were undetectable in all patients, as these genes are not expressed in bone marrow or peripheral blood unless they are deregulated. *LMO2* showed a stable basal expression in all samples. Overall, RNA results were in 100% agreement with the expected results.

A summary of ALLseq performance can be found in Table 2 and Supplemental Table S4.

### 2.4. Limit of Detection, Reproducibility, Repeatability, and Linearity

**Figure 3:**
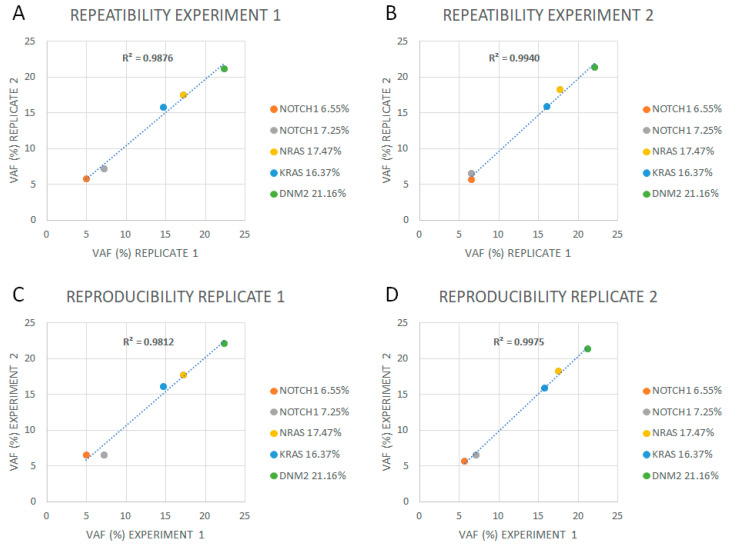
Repeatability and reproducibility of ALLseq. (**A**) Replicates in experiment 1. (**B**) Replicates in experiment 2. (**C**) Replicate 1 analyzed in two different experiments. (**D**) Replicate 2 analyzed in two different experiments.

The limit of detection (LoD) for SNVs and indels was established at 2% of VAF. Intra-experiment repeatability and inter-experiment reproducibility showed a 100% concordance above this VAF, and a high correlation (R^2^ ≥ 0.98) was observed between inter- and intra-sequencing runs (Figure 3, Supplemental Table S6).

The CNV LoD was established at a copy number ratio of 0.5, corresponding with a heterozygous deletion, when, at least, confidence > 20 and precision > 10 were reached (Table 3). Different analysis rounds showed 100% repeatability and reproducibility for CNV analysis. 

Regarding fusion expression quantitation, serial dilutions yielded a linear range up to the 10^−4^ dilution (Supplemental Figure S2).

### 2.5. Clinical Validation: Prospective Sequencing

In total, 43 correlative patients were prospectively analyzed with ALLseq, whose main characteristics are shown in Supplemental Table S7.

DNA sequencing identified 54 SNVs or indels, resulting in a mean of 1.26 variants per sample. Twenty-five cases (58.1%) harbored at least one mutation. The genes with the highest mutational frequency were *KRAS* (18.52%) (10/54), *NRAS* (14.81%) (8/54), *PTPN11* (14.81%) (8/54), and *NOTCH1* (9.26%) (5/54). All of the SNVs and indels were confirmed by direct Sanger sequencing (Supplemental Table S8, Supplemental Figure S3A).

**Figure 4:**
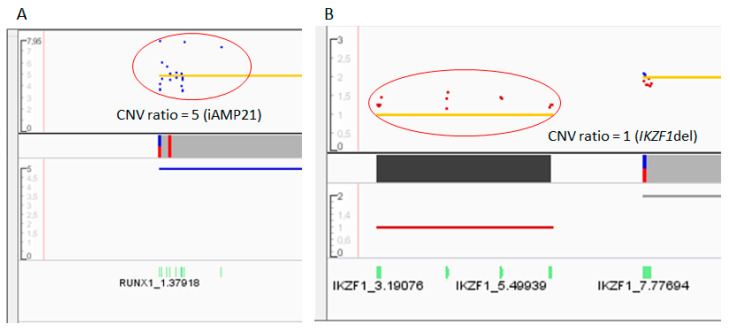
Copy number variations identified by ALLseq in two different patients. The x axis shows the gene regions, while the y axis represents the copy number ratio values. The red circles highlight the gains or losses. (**A**) The dot plot shows a gain of *RUNX1*, associated with the iAMP21 entity. (**B**) The dot plot shows the loss of exons 4–7 of *IKZF1*, resulting in the main pathogenic isoform (IK6) in ALL.

Sixty-eight CNVs were found in 28 out of 43 samples (65.12%). The mean number of affected genes was 1.58 (range 0–6) per patient. The most frequently deleted gene was *CDKN2A* (19.12%; 13/68), followed by *CDKN2B* (16.18%; 11/68) and *ETV6* (8.82%; 6/68). Notably, ALLseq detected additional copies of *RUNX1* and *TP53* in patients carrying chromosome 17 and 21 gains (including a patient harboring intrachromosomic amplification of chromosome 21, iAMP21), respectively (Figure 4, Supplemental Figure S3B). Results from 5/43 (11.63%) patients were discordant with MLPA (Supplemental Table S8).

Overall, a total of 122 SNVs, indels, and CNVs were detected in the DNA. Genes affected by these alterations were grouped according to the signaling pathway in which they have a role. The most frequently altered were the TP53-cell cycle (30.33%; 37/122), followed by the RAS (18.03%; 22/122) and lymphoid differentiation (17.21%; 21/122) pathways. Interestingly, the RAS pathway was only affected by SNVs or indels and represented 41.07% (22/54) of these alterations (Supplemental Figure S4).

ALLseq detected fusions in 11/43 samples (25.6%). The most frequent fusion was *ETV6::RUNX1*, identified in 5/43 patients (11.6%), followed by *KMT2A* rearrangements and *STIL::TAL1*, each detected in 2/43 patients (4.6%) (Supplemental Table S8). All the fusions were confirmed by orthogonal methods.

**Figure 5:**
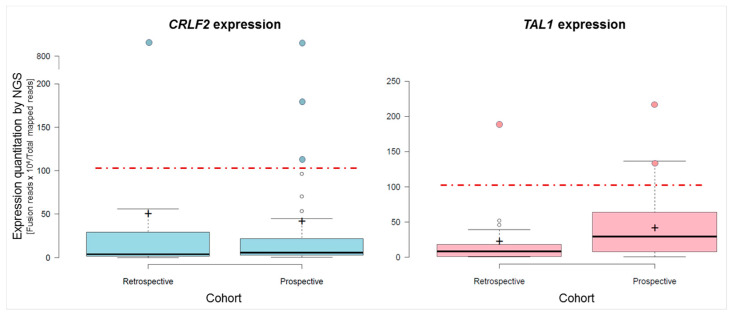
Box and Whiskers plots of *CRLF2* (**left** panel) and *TAL1* (**right** panel) expression, quantified by ALLseq. For each gene, the expression levels on the retrospective and prospective cohorts are depicted. The red discontinuous line marks the overexpression cutoff value of 105 (target reads × 10^4^/total mapped reads). Samples overexpressing the target gene for which the molecular mechanism was confirmed are highlighted as colored dots.

Regarding gene expression, 3 out of 43 (6.9%) patients overexpressed *CRLF2.* One harbored the t(X;14)(p22;q32) translocation (detected by cytogenetics and FISH); another carried the *CRLF2::CSF2RA* fusion, also detected by ALLseq and FISH; and the third patient showed a pseudoautosomic region 1 (PAR1) amplification, which contains *CRLF2.* Furthermore, the two samples harboring *STIL::TAL1* (detected by ALLseq and FISH) overexpressed *TAL1* (Figure 5). Additionally, two patients with translocated *TLX3* (confirmed by FISH) met the overexpression criteria for this gene (Supplemental Table S8).

Of note, we detected ectopic *TLX1* and *NKX2-1* expression in two patients. These genes are not usually expressed in hematopoietic tissue unless deregulated, but *TLX1* was expressed in one T-cell patient harboring the t(7;17)(q31;q12) translocation (identified by conventional cytogenetics), and *NKX2-1* was expressed in a T-cortical patient for which no molecular mechanism was found.

**Figure 6:**
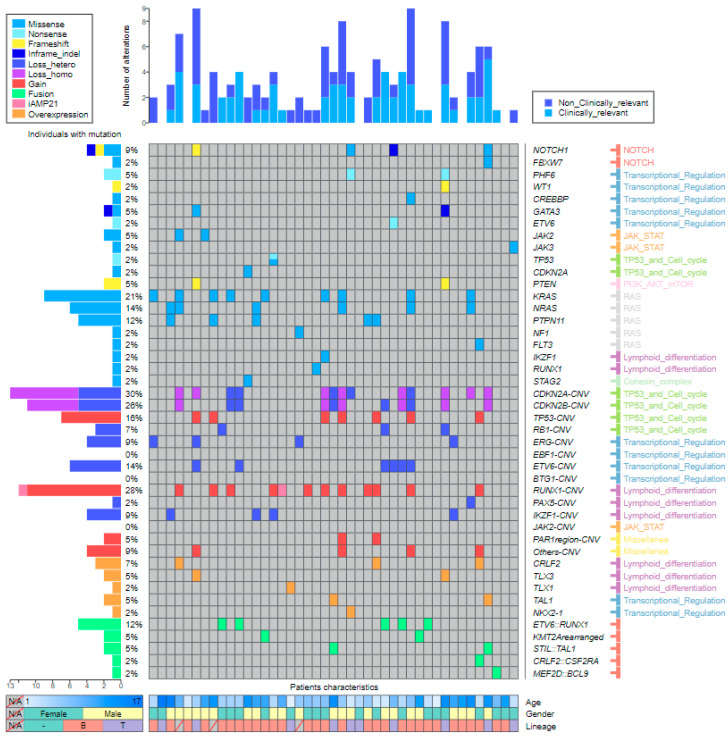
Landscape plot representing ALLseq results. Altered genes are listed at the right side of the panel. Genes are arranged first by the alteration type and second by the involved pathway. The percentage of patients carrying alterations is noted at the left side of the figure. Samples are displayed as columns, with clinical relevance of mutations plotted at the top. The main patient characteristics are shown at the bottom.

Overall, the combined DNA and RNA results showed a total of 142 alterations. Comprehensive molecular and basic clinical data are shown in Figure 6.

Next, we tested the ALLseq clinical yield by exclusively classifying prospective patients according to these NGS results. Only pathogenic variants complying with at least one of these criteria were considered as clinically relevant, i.e., (a) variants that define World Health Organization (WHO 2022) and/or the International Consensus Classification of myeloid neoplasms and acute leukemias (ICC 2022) categories [3,4] (of note, ALL classification was updated during the development of this project so we used the latest classifications, although the design was based on the previous versions); (b) variants defining genetic risk groups considered by the ALLTogether treatment protocol; and (c) variants allowing patient selection for targeted therapy according to the ALLTogether protocol or active clinical trials.

**Figure 7:**
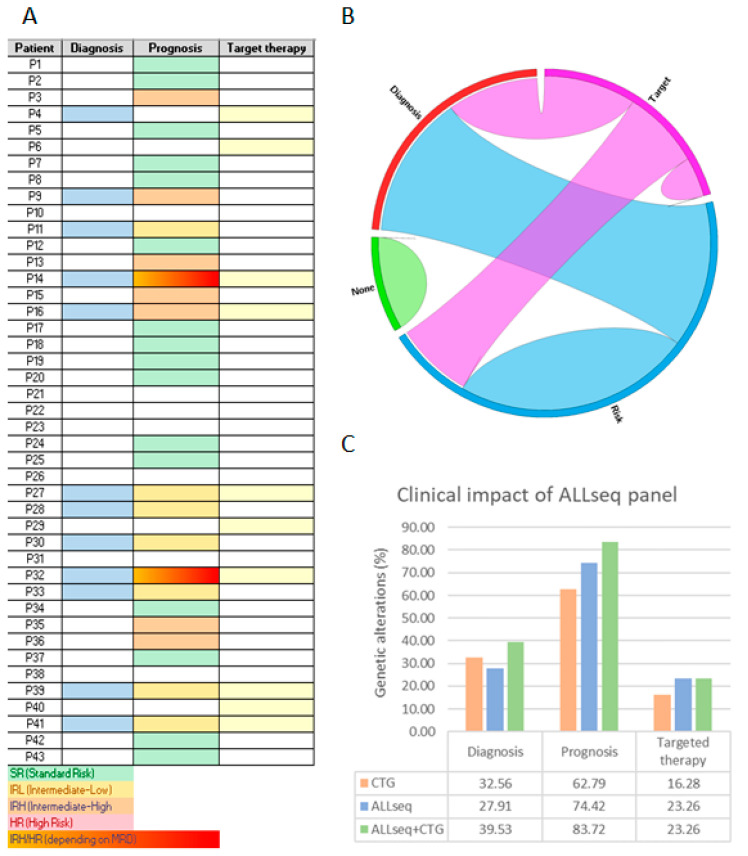
Clinical utility of the molecular results yielded by ALLseq. (**A**) Heat map identifying patients for whom useful alterations for diagnosis, risk stratification, and/or targeted therapy selection was found. (**B**) Circos diagram generated from the heat map data. (**C**) Percentage of patients that can benefit from molecular results when ALLseq and/or cytogenetics (CTG) are used.

Under these premises, 63/142 (44.37%) of the pathogenic variants were considered as clinically relevant. Subsequently, ALL patients were allocated into three groups, depending on the clinical utility of the alteration(s) they carried: diagnosis, risk stratification, and/or targeted therapy. Moreover, 12/43 (27.91%) patients carried entity-defining alterations, 32/43 (74.42%) harbored risk-associated lesions, and 10/43 (23.26%) were suitable for targeted therapy (Figure 7A). Co-occurrence among these categories is shown in Figure 7B. Overall, 36/43 (83.72%) patients could benefit from molecular findings derived from ALLseq. Among the seven remaining patients, four harbored aneuploidies, and three did not show any additional molecular lesion.

Finally, we analyzed the clinical utility of ALLseq and cytogenetics as independent or combined techniques. When used as a stand-alone method, ALLseq provided information related to prognosis or treatment to more patients than cytogenetics, while slightly more patients benefited from cytogenetics than ALLseq for the identification of ALL entities. By combining both techniques, most patients (40/43; 93.02%) could be diagnosed, classified into risk groups, and/or benefit from targeted therapies (Figure 7C).

## 3. Discussion

In the present study, an ALL-targeted NGS panel was designed and validated. ALLseq allows clinically relevant SNVs, indels, CNVs, fusions, and gene expression alterations to be detected, which, to the best of our knowledge, makes our panel unique.

ALLseq was conceived specifically for somatic analysis. However, recent research points to germline variants as a driving mechanism in familiar cases [10]. In suspected cases, germline origin must be confirmed in culture skin fibroblasts according to current recommendations. If confirmed, the patient should be assigned to a specialized unit [11,12].

ALLseq sequencing metrics were equivalent to those reported by commercial panels such as the OCCRA, which were also in line with its Illumina counterpart [13]. The overall performance of ALLseq, as assessed by sensitivity, specificity, PPV, NPV, and accuracy, was 100% on SNVs, indels, and fusions, and only CNV identification was slightly poorer.

The reliable identification of CNVs using NGS continues to be a challenge due to unequal target coverage [14]; however, ALLseq CNV reliability was acceptable according to Cohen’s criterion.

The LoD was set at 2% VAF for SNVs and indels, which is acceptable in the clinical context, as most protocols establish 5% as the cut-off value for considering variants as clinically relevant [15,16]. Although the LoD is sufficient for SNV and indel detection, special attention to low blast % samples is required when analyzing CNV and gene expression with NGS, as recommended by Jennings et al. [17].

The main limitation of ALLseq, like most targeted panels, is its inability to identify aneuploidies, which are present in up to 30% of pediatric ALL cases. However, these alterations are easily identified by cytogenetic techniques, which are routinely performed in all laboratories. It cannot detect *DUX4* deregulation (which represents 7% of B-ALL), which has been considered an independent entity by the WHO 2022 and ICC 2022 classifications and confers favorable prognosis [18]. *DUX4* deregulation is technically difficult to detect, as it is located within a repetitive region on chromosome 4q, with an almost identical locus on 10q. Thus, primers can bind to multiple loci on both 4q and 10q. Additionally, *DUX4* fusions show great variability in breakpoints [19].

ALLseq allows 634 fusions to be identified (including ABL-class translocations and virtually all of the class-defining fusions), as well as the aberrant expression of seven genes including *CRLF2*. Notably, up to 50% of Ph-like ALLs overexpress this gene due to fusions, mutations, and alterations in the JAK-STAT pathway [20]. Therefore, with the ALLseq design, most Ph-like patients can be diagnosed, allowing the identification of candidates for targeted therapies with tyrosine kinase or JAK-STAT inhibitors (NCT03571321). In fact, we were able to define the ALL subtype of around 30% of patients, among whom 7% were Ph-like cases overexpressing *CRLF2*.

The recent update of the WHO 2022 and new ICC ALL classifications included, for the first time in this disease, categories defined by point mutations. Moreover, the potential use of targeted therapies in patients carrying SNVs or indels highlighted the clinical relevance of these alterations in ALL. In fact, ALLseq identified point mutations in FLT3, the *NOTCH1* pathway, or *JAK* family genes, for which targeted inhibitors have been developed [21], in 16% of patients.

Regarding CNV identification, *IKZF1* deletions have been classically recognized as conferring poor prognosis [22]. More recently, several European groups have developed different CNV profiles that are significant for risk stratification. In particular, the COALL has proposed the *IKZF1plus* group, defined by the deletion of *IKZF1* co-occurring with at least one additional deletion in *CDKN2A/B*^homo^, *PAX5*, or the pseudo autosomic region 1 (PAR1) in the absence of *ERG* deletion, which distinguishes high-risk ALL patients who benefit from treatment intensification [23]. Similarly, the British group proposes a CNV profile (UKALL-CNA) involving *IKZF1*, *CDKN2A/B, PAR1, BTG1, EBF1, PAX5, ETV6*, and *RB1* to refine risk groups [24]. The ALLTogether (NCT04307576) treatment protocol incorporates the UKALL-CNA risk stratification, making CNV assessment mandatory. With ALLseq, we were able to correctly risk-stratify around 90% of patients.

Cytogenetic approaches have been the main diagnostic tool in ALL, given the exclusive importance of aneuploidies and a few translocations in this disease just a decade ago [25]. However, the development of “omic” technologies has substantially broadened the spectrum of molecular lesions that explain the onset of ALL. In this context, NGS has been incorporated as a complementary tool into most clinical laboratories [26]. Our results show that the combination of ALLseq and cytogenetics provide clinical information to virtually all patients, reducing the number and type of assays necessary for ALL characterization (RT-PCR, MLPA, SNP arrays, etc.). It is worth mentioning emerging technologies, such as optical genome mapping, which will surely be useful for the analysis of ALL patients. This technique, unlike NGS targeted panels, is not restricted to a list of genes and therefore is able to detect novel alterations [27]. Moreover, it can detect numerical and structural chromosome alterations as well as gene-level gains or losses, which makes it very useful for ALL characterization. However, it is unable to detect SNVs and indels which, as discussed above, are currently needed for ALL diagnoses [3,4]. The continuous availability of novel genomic methodologies makes it difficult to define the optimal technology(ies) for ALL characterization. The molecular knowledge of ALL is an evolving field; therefore, the best diagnostic approach has to be flexible and adapt to conform to the latest guidelines. In this context, interdisciplinary groups carrying out an integrated diagnosis become essential in configuring the diagnostic workflow of ALL [28].

In conclusion, ALLseq allows the most frequent alterations in ALL to be identified. Although there are certain limitations to be considered when interpreting the results, the panel constitutes a useful tool for patient characterization and management, as it allows the identification of driver and secondary alterations in a single experiment, thus permitting accurate diagnosis, patient risk stratification, and (in some cases) treatment selection.

## 4. Materials and Methods

### 4.1. Patient Samples and Inclusion Criteria

The study included pediatric and adolescent (≤18 years old) ALL patients diagnosed at Hospital Universitari i Politècnic La Fe (Valencia, Spain). Inclusion criteria were as follows: availability of high-quality DNA and RNA from bone marrow or peripheral blood, and written informed consent in accordance with the recommendations of the Declaration of Human Rights and the Conference of Helsinki. The Institutional Ethics Committee for Clinical Research approved this study (approval numbers 2021-045-1 and 2022-09-04).

### 4.2. ALLseq: An ALL-Targeted Custom NGS Panel

A custom panel targeting ALL (ALLseq) was designed using the White Gloves Service from Thermo Fisher Scientific. Target selection was based on its potential clinical utility according to 2 levels of evidence:

- Level 1: clinical guidelines and clinical trials: (a) alterations included in the WHO classification of hematolymphoid tumors in force at the time of the start of the study [29]; (b) alterations defining genetic ALL subtypes [30]; (c) alterations used for risk stratification by international cooperative groups [31], NCT04307576]; (d) alterations used for potential targeted therapy [32].
- Level 2: other pathogenic alterations described in large cohorts: (a) variants that cluster into specific subtypes of ALL [15,33]; (b) variants associated with good or bad prognosis but not currently used for patient risk stratification [34]; (c) variants that confer resistance to specific drugs in vitro/in vivo experiments [35].

The sequencing workflow was carried out on Ion Torrent platforms (Thermo Fisher Scientific, San Francisco, CA, USA). DNA libraries were generated from 10 ng of DNA, with an initial PCR consisting of 17 cycles and 4 min of extension time; for RNA libraries, cDNA was generated with the SuperScript™ IV VILO™ kit (Thermo Fisher Scientific) from 10 ng of total RNA, and PCR was performed with 20 cycles and 4 min of extension time. Library and template preparation was carried out automatically on the Ion Chef™ Instrument (Thermo Fisher Scientific) using the Ion AmpliSeq™ Kit for Chef DL8 (Thermo Fisher Scientific) and the Ion 510™ & Ion 520™ & Ion 530™ Kit-Chef (Thermo Fisher Scientific), respectively. Libraries from eight samples were loaded onto an Ion 530™ Chip (Thermo Fisher Scientific) and sequenced on an Ion S5 sequencer (Thermo Fisher Scientific).

### 4.3. Data Analysis

Human genome build 19 was used as the reference genome. Base calling was performed on Torrent Suite software version 5.10.0 (Thermo Fisher Scientific). Variant identification was accomplished with the Variant Caller Plugin (Thermo Fisher Scientific), and variant annotation was performed using Ion Reporter (IR) software version 5.10.3.0 (Thermo Fisher Scientific).

For CNV assessment, the normalized read depth of each sample was compared with that of the reference baseline (generated by sequencing 20 healthy controls). The IR software applied an algorithm based on a hidden Markov model, which predicts the copy number or the ploidy state. A copy number of 2 was considered as normal, values ≥ 3 were considered as amplifications, and a copy number ratio of one or zero suggested heterozygous or homozygous deletions, respectively.

### 4.4. ALLseq Validation Strategy

For technical validation, 25 retrospective patients harboring a >90% blast count in bone marrow or peripheral blood and a complete molecular characterization were selected. A second validation round was carried out by sequencing sequential unbiased ALL samples to assess the clinical utility of the panel.

#### Complementary Molecular and Cytogenetic Methods

All the ALL samples were analyzed at diagnosis by the following methods: conventional cytogenetics, an ALL FISH custom panel (Cytocell Ltd., Cambridge, UK), RT-PCR to asses *ETV6::RUNX1* and *BCR::ABL1* fusions [36], and MLPA SALSA P335 ALL-IKZF1 (MRC Holland, Amsterdam, NL).

The Oncomine Childhood Research Assay (OCCRA; Thermo Fisher Scientific) was used retrospectively to further characterize ALL samples following the manufacturer’s instructions. After sequencing, variant filtering was performed on IR software version 5.10.3.0 (Thermo Fisher Scientific). For ALLseq technical validation, intronic and synonym variants were filtered out, whereas pathogenic and likely pathogenic variants, as well as variants of unknown significance (VUS), were retained.

Variants detected by NGS (OCCRA and/or ALLseq) were confirmed by Sanger sequencing and Minor Variant Finder software (Thermofisher Scientific) (SNVs and indels) or qRT-PCR (fusions and gene expression alterations). Fusion characterization and gene expression were assessed by qRT-PCR on a LightCycler 480 II (Roche Diagnostics, Switzerland, AG) using Sybr green and *ABL1* as the control gene. Primers and PCR parameters are described in Supplemental Tables S9 and S10. For selected samples, optical genome mapping (OGM) was used following the manufacturer’s instructions in order to confirm CNVs not included in the MLPA SALSA P335 ALL-IKZF1.

### 4.5. ALLseq Technical Validation

#### 4.5.1. Run Metrics and Quality Criteria

A DNA result was considered evaluable if it met the following requirements: mean read depth ≥ 1500× per sample; uniformity and on-target reads ≥ 80%; and MAPD < 0.5, confidence > 20, and precision > 10 for CNV analysis. Regarding RNA, a minimum number of 20,000 mapped reads was established; gene and fusion expression levels were calculated as [(target reads × 1000)/total RNA reads].

The mean on-target and uniformity percentages, depth of coverage, and MAPD yielded by ALLseq were compared with those from the OCCRA.

#### 4.5.2. Assessment of Analytical Performance

Sensitivity [true-positive (TP)/(TP + false-negative (FN))], specificity [true negative (TN)/(TN + false-positive (FP))], precision [(TP + TN)/n], positive predictive values (PPVs) [TP/(TP + FP)], and negative predictive values (NPVs) [FN/(FN + TN)] were assessed by comparing ALLseq results with data from orthogonal techniques described above. The Bland–Altman method was used to assess the variant allele frequency (VAF) agreement between ALLseq and OCCRA panels. CNV detection reliability was further evaluated with Cohen’s kappa coefficient, where a value > 0.8 indicates a high agreement with the gold-standard method.

Fusion expression linearity was assessed by diluting an *ETV6::RUNX1*-positive sample at 1:10, 1:100, and 1:1000 ratios into a negative control. Gene expression was quantified by ALLseq and qRT-PCR and compared. The overexpression cutoff was established at 105 expression units (target reads × 10^4^/total mapped reads). In order to calculate analytical performance for gene expression, expression units were dichotomized (overexpression vs. no overexpression) according to the cutoff criteria.

#### 4.5.3. Limit of Detection, Reproducibility, and Repeatability

To assess the LoD, repeatability, and reproducibility, a DNA pool from samples harboring *NOTCH1* (p.Phe2509fs, VAF 6.55%; p.Phe1606_Lys1607insAspSerPro, VAF 7.25%)*, NRAS* (p.Gly12Cys, VAF 17.47%)*, KRAS* (p.Leu19Phe, VAF 16.37%), and/or *DNM2* (p.Arg123Ter, VAF 21.16%) was created. Two serial VAF dilutions (ratios of 1:2 and 1:4) were prepared using a wild-type control sample. For each dilution, two independent libraries were sequenced twice in back-to-back experiments. In these experiments, a coefficient of variation (CV) ≤ 20% was considered acceptable for VAF values.

In order to obtain the CNV LoD, two samples harboring *CDKN2A/B* homozygous deletion (*CDKN2A/B*^homo^) and *IKZF1* heterozygous deletion (*IKZF1*^hetero^), respectively, were combined at different ratios (*CDKN2A/B*^homo^:*IKZF1*^hetero^; 3:1, 1:1, and 1:2) creating different copy number ratios. A sample pool was analyzed in three consecutive experiments to test the reproducibility and repeatability.

### 4.6. ALLseq Clinical Validation

A total of 43 correlative patients were prospectively analyzed. In these patients, pathogenic and likely pathogenic variants were confirmed with complementary methods, as described above.

### 4.7. Statistical Analysis

Medians of quantitative variants were compared with Mann–Whitney’s U test. Qualitative parameters were compared with the chi-square’s test and Cohen’s kappa coefficient. A Bland–Altman plot was used to assess the agreement between VAF values obtained by ALLseq and OCCRA panels. Box plots were generated with BoxPlot R (http://shiny.chemgrid.org/boxplotr, accessed on 16 December 2022). A Circos plot was generated as described by Krzywinski et al. [37]. A landscape diagram was created using Oviz-bio, a free web-based platform for interactive data visualization [38].

## Figures and Tables

**Table 1:** Main targets included in the ALLseq design.

DNA (2 Primer Pools): 96,7 kb; 1138 Amplicons, 97.91% Coverage		RNA (2 Primer Pools)	
HotSpots (SNVs and Indels; 22 Genes)	Whole Coding Sequence (SNVs and Indels; 32 Genes)	Fusions (271 Fusions, 634 Isoforms)	Expression Quantitation (7 Genes)
CREBBP *, CRLF2, DNMT3A, EP300, EZH2, FBXW7, FLT3, IDH1/2, IL7R, JAK1/2/3, NOTCH1, PAX5, PIK3CA, PTPN11, K/NRAS, SETD2, STAT5B, and SH2B3 *	AKT, BCL11B, BTG1, CDKN2A/B *, DNM2, EBF1, EED, ERG *, ETV6 *, GATA3, IKZF1 *, IL2RB, KDM6A, LEF1, NF1, NT5C, PAX5 *, PHF6, PTEN, PTPN2, RB1 *, RUNX1 *, STAG2, SUZ12, TET2, TP53, and *WT1*	Main drivers: *ABL1/2, KMT2A, TCF3, ETV6, EPOR, CSF1R, FLT3, JAK2, PDGFRA/B, LYN, NTRK1/2/3, TYK2, FGFR1, IL2RB, TSLP, PAX5, NOTCH1, MEF2D, ZNF384,* and *MYB/L1*. Main fusions: *P2RY8::CRLF2, SET::NUP214, PICALM::MLLT10,* and *STIL::TAL*	*CRLF2, HOXA, LMO2, NKX2, TAL1, TLX1,* and *TLX3*

* CNV can be assessed.

**Table 2:** Main ALLseq analytical performance.

Alteration Type	Sensitivity	Specificity	PPV	NPV	Precision
SNVs and indels	100%	100%	100%	100%	100%
CNVs (Cohen kappa coefficient = 0.88)	88.87%	97.92%	92.85%	95.91%	96.45%
Fusions	100%	100%	100%	100%	100%
Gene expression	100%	100%	100%	100%	100%

**Table 3:** Limit of detection for CNVs.

	Copy Number Ratio			
	*CDKN2A/B*		*IKZF1*	
	Expected	Observed	Expected	Observed
Dil1 (3:1)	0.5	<1	0.25	ND ^1^
Dil2 (1:1)	1	1	0.5	<1
Dil3 (1:2)	0.66	<1	0.66	<1

^1^ ND = not detected.

## Data Availability

All the data and materials are available upon reasonable request to llop_margar@gva.es.

## Supplementary Materials

The supporting information can be downloaded at: https://www.mdpi.com/article/10.3390/ijms24054440/s1.
