# Supplementary material for: Design and Validation of a Custom Next-Generation Sequencing Panel in Pediatric Acute Lymphoblastic Leukemia
Source: Int J Mol Sci. 2023 Feb 23;24(5):4440. doi: 10.3390/ijms24054440 (PMC10002321; doi:10.3390/ijms24054440)
Supplement: Supplementary file 1 [file ijms-24-04440-s001.zip › Supplemental Figures_DEF.pdf]

Supplemental Figures

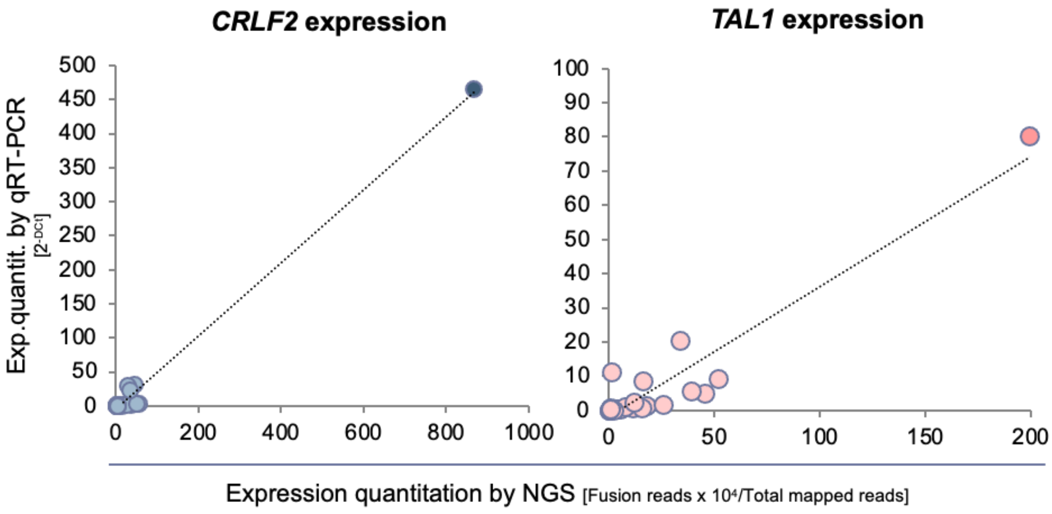

Supplemental Figure S1. Gene expression quantitation of *CRLF2* (left panel) and *TAL1* (right panel) measured by NGS and qRT-PCR.

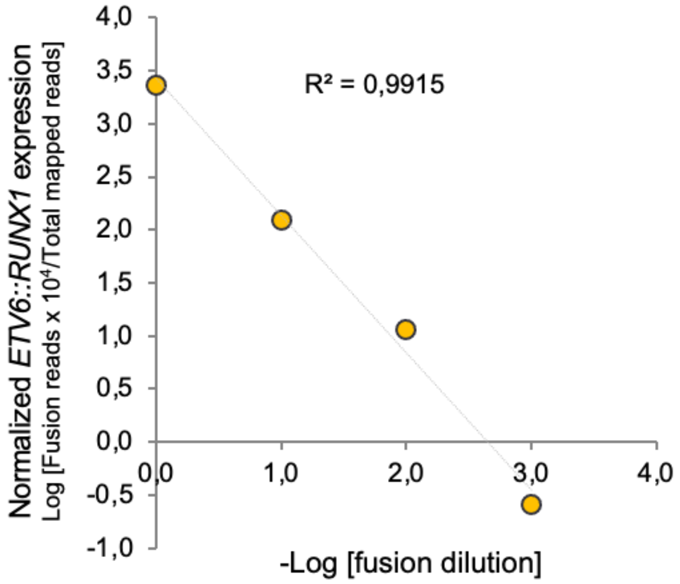

Supplemental figure S2. Assessment of ALLseq fusion expression linearity

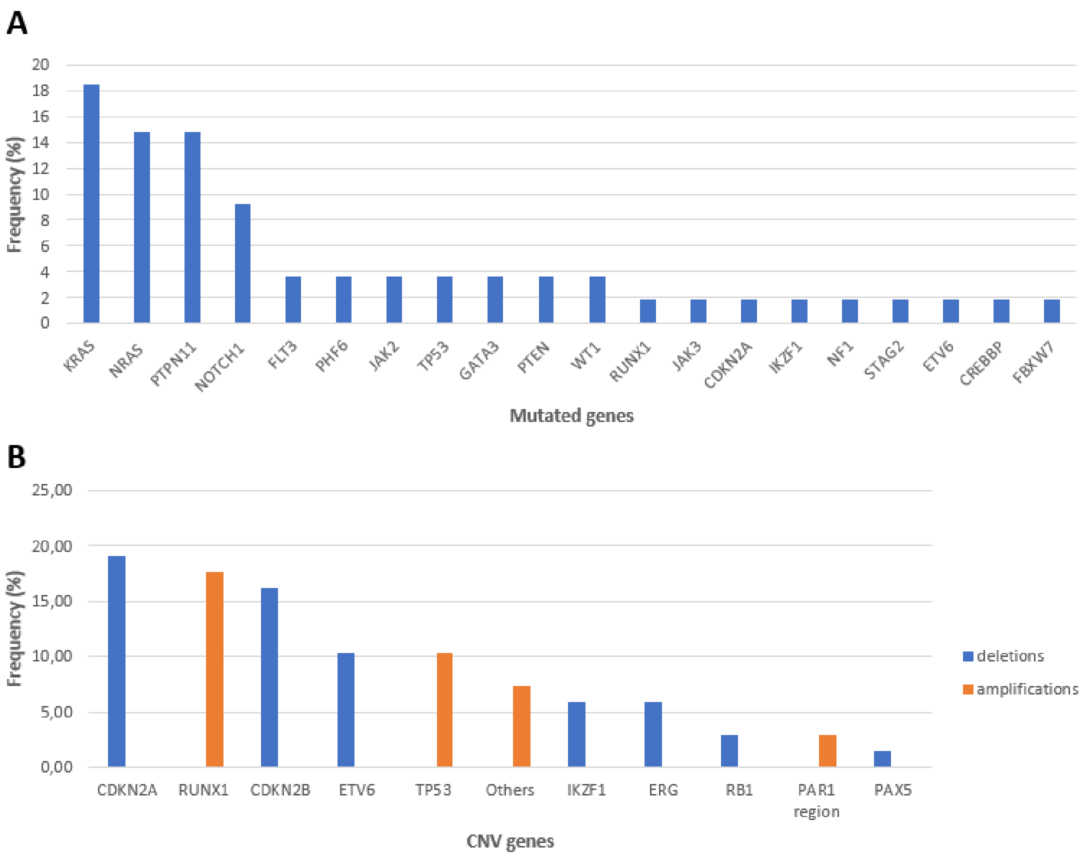

Supplemental Figure S3. DNA results from the prospective cohort. A) Genes affected by SNVs or indels. B) Genes affected by CNVs

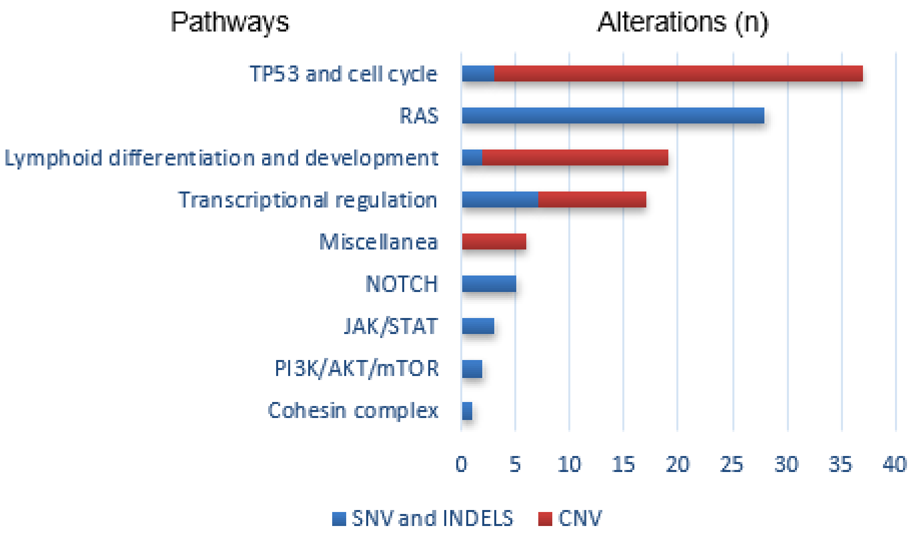

Supplemental Figure S4. Number and type of DNA alterations, detected by ALLseq, affecting the main signaling pathways in ALL
